# Ganglionated Plexi Ablation for the Treatment of Atrial Fibrillation

**DOI:** 10.3390/jcm9103081

**Published:** 2020-09-24

**Authors:** Sahar Avazzadeh, Shauna McBride, Barry O’Brien, Ken Coffey, Adnan Elahi, Martin O’Halloran, Alan Soo, Leo. R Quinlan

**Affiliations:** 1 Physiology and Human Movement Laboratory, CÚRAM SFI Centre for Research in Medical Devices, School of Medicine, Human biology building, National University of Ireland (NUI) Galway, H91 TK33 Galway, Ireland; sahar.avazzadeh@nuigalway.ie (S.A.); shauna.mcbride@nuigalway.ie (S.M.); 2 AtriAN Medical Limited, Unit 204, NUIG Business Innovation Centre, Upper Newcastle, H91 TK33 Galway, Ireland; barry.obrien@atrianmedical.com (B.O.); ken.coffey@atrianmedical.com (K.C.); 3 Translational Medical Devise Lab (TMD Lab), Lambe Institute of Translational Research, University College Hospital Galway, H91 ERW1 Galway, Ireland; adnan.elahi@nuigalway.ie (A.E.); martin.ohalloran@nuigalway.ie (M.O.); 4 Electrical & Electronic Engineering, School of Engineering, National University of Ireland Galway, H91 TK33 Galway, Ireland; 5 Department of Cardiothoracic Surgery, University Hospital Galway, Saolta Hospital HealthCare Group, H91 YR71 Galway, Ireland; Alan.Soo@hse.ie

**Keywords:** atrial fibrillation, ganglionated plexi, autonomic nervous system, ablation

## Abstract

Atrial fibrillation (AF) is the most common type of cardiac arrhythmia and is associated with significant morbidity and mortality. The autonomic nervous system (ANS) plays an important role in the initiation and development of AF, causing alterations in atrial structure and electrophysiological defects. The intrinsic ANS of the heart consists of multiple ganglionated plexi (GP), commonly nestled in epicardial fat pads. These GPs contain both parasympathetic and sympathetic afferent and efferent neuronal circuits that control the electrophysiological properties of the myocardium. Pulmonary vein isolation and other cardiac catheter ablation targets including GP ablation can disrupt the fibers connecting GPs or directly damage the GPs, mediating the benefits of the ablation procedure. Ablation of GPs has been evaluated over the past decade as an adjunctive procedure for the treatment of patients suffering from AF. The success rate of GP ablation is strongly associated with specific ablation sites, surgical techniques, localization techniques, method of access and the incorporation of additional interventions. In this review, we present the current data on the clinical utility of GP ablation and its significance in AF elimination and the restoration of normal sinus rhythm in humans.

## 1. Introduction

Atrial Fibrillation presents clinically as chaotic electrical excitation that is detrimental to normal atrial contractility [1]. AF is the most common form of cardiac dysrhythmia and is categorized as a supraventricular tachyarrhythmia, which will affect 18 million people in Europe and 6–12 million in the United States by 2060 and 2050, respectively [2,3,4,5]. AF is generally classified as either paroxysmal, persistent, or long-standing persistent, and its presentation can in fact evolve and change over time [6]. The effects of AF can be life-threatening, as insufficient contraction of the atria results in blood stasis which promotes the formation of thromb-oemboli which effect the heart but can also propagate to other vital organs [7,8]. Despite many advances in recent years, no specific etiological factor has been pinpointed as the main cause of AF. Some epidemiological and clinical factors such as abnormalities associated with metabolism, endocrine function and genetics, are known to predispose patients to AF [6,9]. Furthermore, pathophysiological factors such as electrical and structural remodelling, inflammation, and local autonomic system regulation are also seen with AF [10]. Evidence from the literature highlights the role of the intrinsic and extrinsic autonomic nervous system (ANS) in cardiac function, the underlying mechanism of altered electrical activity in AF is not fully understood [11]. Altered autonomic activity is recognised as a significant component in both the initiation and maintenance of AF [12,13]. The incidence of atrial arrhythmias is reported to reduce when ANS innervation is significantly decreased [14,15]. The activity of the intrinsic cardiac ANS is found to be disrupted in cases of AF, with studies associating vagal interference with networks of GPs [16,17]. GPs are normally found in close proximity with epicardial fat pads and reside in discrete locations on the atria and ventricles, particularly surrounding the pulmonary veins (PV) and great vessels [18]. Numerous trials employing a variety of therapeutic interventions for cardiac disease have been completed to date, with some targeting GPs for AF treatment. The complex anatomical layout and physiological interconnectivity of these GP sites is important in understanding the pathophysiology of AF [19]. Our aim is to address the association of GPs with AF and document the extant literature reporting the impact of GP ablation procedures recorded in human clinical studies.

## 2. Cardiac Autonomic Nervous System

**Figure 1:**
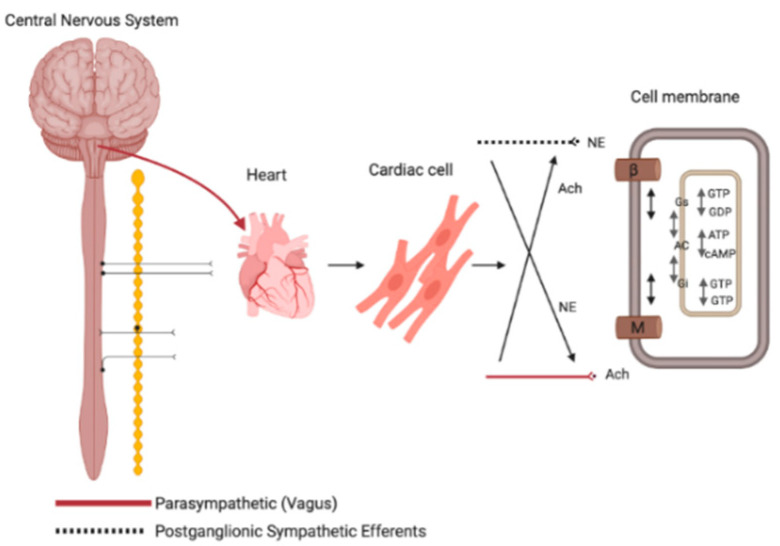
Sympathetic and parasympathetic mechanism in the autonomic nervous system (ANS). Parasympathetic vagal activity (in red) inhibits noradrenaline (NE) which in turn inhibit acetylcholine release (ACh). Released ACh binds to M muscarinic receptors (M) and, through the inhibition of Protein G1, coupled to adenylate cyclase (AC). Released NE from postganglionic sympathetic nerve endings (dotted line) binds to β-adrenergic receptors (β) which activate AC increasing intracellular cyclic-AMP (cAMP).

Components of the peripheral, central and intrinsic cardiac innervation systems form a complex interconnected network that manages cardiovascular function [19,20]. The cardiac ANS is organised into extrinsic and intrinsic components that are supplied by the autonomic nerves. The intrinsic ANS is comprised of clusters of neurons known as GPs that interconnect not only to the atria and ventricles, but also to the extrinsic cardiac ANS. The extrinsic sympathetic innervation arises in the grey matter of the thoracic spinal cord segments T1–T6 and are generally myelinated fibres, that increase heart rate and myocardial contractility by releasing noradrenaline, stimulating inotropy in the heart [18,20] (Figure 1). Noradrenaline (NE) binds to β1-adrenoceptors increasing sodium permeability, thereby increasing heart rate [20]. Parasympathetic fibres arise in the medulla oblongata, pons and midbrain of the brainstem, with some fibres arising from the sacral portion of the spinal cord (S2–S4). The resting heart is dominated by parasympathetic tone, which acts to reduce heart rate and slow cardiac impulses from the atria to the ventricles (Figure 1) through the release of acetylcholine (ACh).The binding of ACh to G-protein coupled muscarinic receptors (M2) activates inhibitory G proteins, reducing both the rate of depolarization and force of contraction of the atria [20]. This is achieved by reducing intracellular cyclic-AMP (cAMP) formation, reversing sympathetic effects on ion channels and Ca^2+^ handling.

### Role of the ANS in the Pathogenesis of AF

Experimental and clinical studies have reinforced the important role of the ANS in AF pathogenesis, initiation and maintenance [12]. Factors including alteration of ion currents, atrial myocardial metabolism and local autonomic regulation are responsible for the multifactorial induction of AF [21]. Reports show that pulmonary vein (PV) focal firing and AF can arise by GP stimulation at the PV-atrial junctions [22,23]. Less commonly, focal initiation of AF can be seen related to ectopic activity from the muscular sleeves of the Superior vena cava (SVC), ligament of Marshall, or regions elsewhere on the left and right atria which possibly coincide with GPs in those regions [24].

Changes in autonomic tone prior to AF onset have commonly been noted [25]. The underlying mechanism behind this is the effect of inward Ca^2+^ and/or outward K^+^ current and the shortening of action potential duration observed in patients with paroxysmal AF [26]. Cervical vagal stimulation causes the release of ACh which activates outward K^+^ currents in atrial myocytes, substantially shortening the action potential duration [27,28]. This has been proven to facilitate the onset and maintenance of AF in patients [29,30,31]. In addition, direct stimulation of GPs is commonly followed by hyperactivity and excess secretion of neurotransmitters, creating ideal conditions for AF initiation and continuation [32]. Excess release of ACh and catecholamines has been shown to result in rapid electrical firing of GPs from both PV and non-PV sites [32,33]. Studies by Po et al. investigated the effects of ACh directly injected into GPs in a canine model of AF and showed it to induce focal firing of PVs and sustained AF [23]. Thus it appears that GP stimulation not only triggers AF in patients, but also directly impacts atrial conduction properties [34]. This influence stems from both sympathetic and parasympathetic branches of the ANS, with the parasympathetic appearing as the predominant branch [34]. GPs provide a site for AF maintenance as autonomic activity was found to increase firing in six-hour rapid atrial pacing recorded from the right anterior GP, showing a decrease in the effective refractory period [35]. A shortening of atrial refractory period (AERP) is commonly seen in AF or rapid atrial pacing [36]. In a canine models of AF, GP ablation reversed electrical remodelling, implying that GP ablation may prove to be a promising strategy for the management of AF in patients [37].

## 3. Ganglionated Plexi

**Figure 2:**
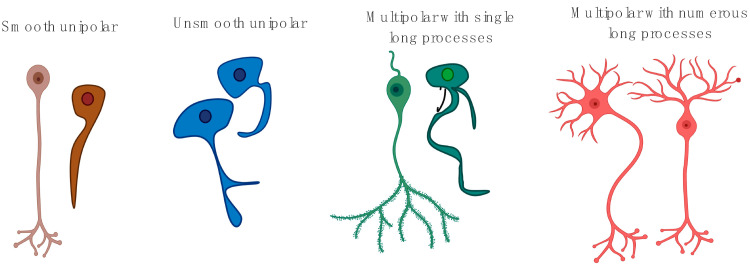
Different morphology of neurons found in ganglionated plexi (GP) sites in humans. There are three types of neurons that are populated in GP sites. These are either unipolar (brown, blue) or multipolar (green, red) having either single (brown, blue, green) or multiple (red) processes.

GPs are localised neural clusters of intrinsic cardiac ganglia, containing local circuits, parasympathetic neurons, and sympathetic afferent and efferent [38]. The variety of neuronal contributions associated with each ganglion reflects their complex synaptology [39]. GPs typically contain 200–1000 neurons and are variable in size, with predominantly oval-shaped soma [17,40]. Histological studies show the mean area of a human ganglia to be 0.07 ± 0.02 mm^2^, with few exceeding 0.2 mm^2^ [41]. Neurons within GPs vary in their projection orientation (unipolar, multipolar) (Figure 2), neurochemical profiles, and abundance on the atria (approx. 400 per GP) and ventricles (approx. 5–40) [40,42,43].

**Figure 3:**
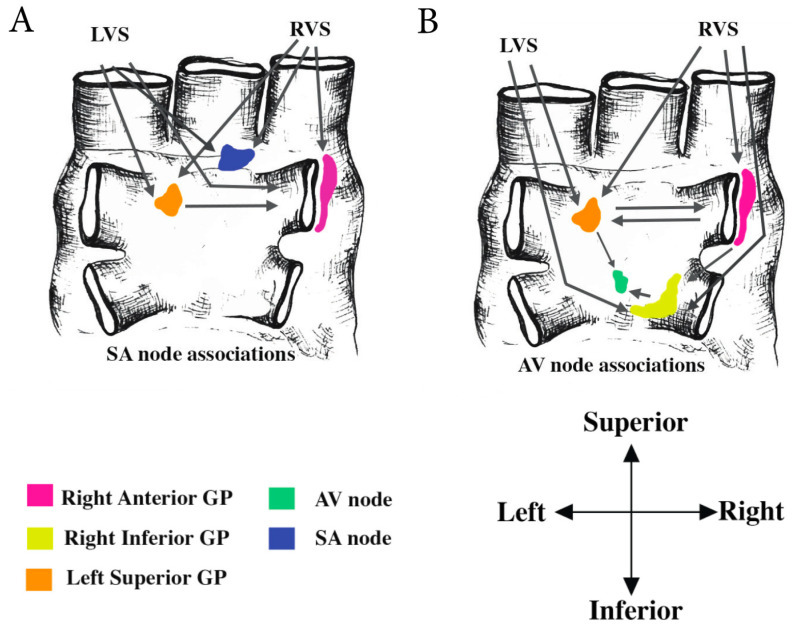
Posterior view of the atria showing the interactions between neural pathways. (**A**): Arrows indicate the direction of impulse and the connections of the left vagosympathetic trunk (LVS) and right vagosympathetic trunk (RVS) with the anterior right (AR) and superior left (SL) GPs. These pathways have been shown to modulate the effects of the sinoatrial node (SA) node and attenuate sinus rate slowing. (**B**): Arrows indicate the direction of impulse and connections involving the LVS and RVS trunks, with the SL, AR and inferior right (IR) GP’s. These pathways have been shown to influence atrioventricular node (AV) node function and ventricular rate response. The inferior left GP (ILGP) acts as a pivotal element in the connection pathway to the AV node.

GPs are typically found embedded in epicardial adipose tissue (EAT) and have been described as having a ‘raisin in bread’ pattern, forming chain-like extensions onto the atria and ventricles [44]. The degree of EAT coverage varies in quantity and depth, and is generally concentrated along the coronary sulcus and interventricular and atrioventricular grooves [45,46]. The electrophysiological characteristics of three distinct epicardial fat pads have been investigated previously. These are located at the intersection of the right atrium and right superior PV (Right Pulmonary Vein-RPV fat pad), the junction of the left atrium and IVC (IVC-LA fat pad), and between the root of the aorta and SVC (SVC-Ao fat pad) superior to the right pulmonary artery [47,48,49] (Figure 3).

### Anatomical Location of GPs

**Figure 4:**
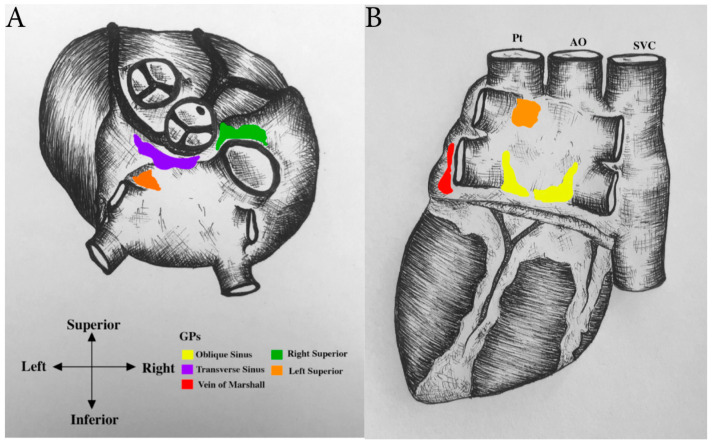
GP’s targeted for ablation. A posterior view of the heart showing all the main ganglionated plexi sites essential for ablation. (**A**) These include the transverse sinus (in purple) and superior right (in green) located between aorta (AO) and superior vena cava (SVC). (**B**) The oblique sinus GPs (in yellow) are situated supero-anterior to the right superior pulmonary vein (PV) (ARPV) and infero-posterior region of the right inferior PV (IRPV). The left superior (in orange) can be found medial to the left superior PV (SLPV). Vein of Marshall (in red) is another target ablation region within the ligament of Marshall. Pt, pulmonary trunk.

In general, GP locations are concentrated on the posterior regions of the atria and the posterior-superior aspect of the ventricles [39] (Figure 3). Knowledge of GP location and their axonal projection pathways are important when considering targeted therapeutic interventions. GPs are found in the posterior portion of the left and right atria (Figure 4), termed the dorso-atrial region, and at the transition from atria to ventricle at the level of the tricuspid and bicuspid valves, in the annular-ventricular region. They are also found around the aorta and pulmonary trunk in the peri-great vessel region, and between the aorta and superior vena cava in the aorto-caval region [50,51]. It is estimated that 75% of epicardial ganglia reside on the dorsal aspect of the heart [41].

There are four GP’s found in the vicinity of the PVs that are regularly targeted in ablation procedures [52]. Each of these GPs innervate the PVs and the surrounding left atrial myocardium [52]. The superior left GP (SLGP) is located superolateral and medial to the left superior PV and extends around its root [16,44,53] (Figure 3). The SLGP is associated with both the sino-atrial (SA) and atrio-ventricular (AV) nodes, influencing sinus and ventricular rate [54,55]. The anterior right (AR) GP is situated supero-anterior to the right superior PV [52]. This GP has been found to have the most prominent interconnections converging with the SA node, where it acts as an integration center connecting the extrinsic ANS to the SA node [52]. The inferior left GP (ILGP) is located infero-posterior to the left inferior PV and has electrophysiological associations with the AV node, and can induce ventricular slowing caused by left vago-sympathetic stimulation [55]. Finally, the inferior right (IR) ganglion can be found in the infero-posterior region of the right inferior PV. The right inferior ganglion has associations with the AV node [52,53,54,56]. Together, the ILGPs and IRGPs are retro-atrial and termed the Oblique Sinus GPs [50].

The circuitry connecting the PV ganglia can be categorized according to the region first in contact with the vago-sympathetic trunk [54]. They can be separated into three individual pathways, with the SLGP linked to one circuit and the ARGP linked to two. The neural route, the right vago-sympathetic trunk-ARGP-SA node, is the predominant pathway and is linked to the left and right vago-sympathetic trunks where it modulates sinus rhythm and shortens the atrial refractory period, without disrupting the AV node [54,57] (Figure 3). The IRGP acts primarily on the AV node, and influences ventricular rate slowing responses induced by vago-sympathetic stimulation [54,56]. Ablation studies have shown that the SLGP does not augment sinus rhythm, but stimulation by the AR, IL and IR GPs cause an increase in rate [57].

The morphology of PVs has also been an area of interest to electrophysiologists. As the vein approaches the ostium, atrial tissue doubles over its circumference forming a fragmented myocardial sheath of pacemaker cardiomyocytes and multilayer muscles [58,59]. These myocyte layers are arranged in bundles that are predominantly spiral and circularly orientated [60]. They often associate with other bundles forming a ‘mesh-like’ assortment of longitudinal and oblique fibers. Ectopic foci have been found to emanate from PVs that can fire at random and induce atrial depolarization [60,61]. A number of groups have shown that the PV sites and the junction between the PVs and left atrium are abundantly enriched with innervation from both sympathetic and parasympathetic nerves, which may contribute to the frequent disruption of signals by GPs in the vicinity [59,62,63]. Experimental and clinical evaluations from some studies have suggested that the formation of spontaneous electrical discharges from PV sites is the result of abnormal automaticity, triggered activity or micro re-entry of electrical signals [60]. Other reports suggest the triggering to be due to PV-associated ganglia rather than the PV itself [64]. An influx of ACh has been touted as central to the stimulation of PV ganglia, inducing PV firing by re-entry pathways in other works [65]. The effect of Ach is to reduce action potential duration in the PV sleeves, causing them to fire until suppressed. Therefore, elimination of PV trigger sites by ablation of the associated ganglia removes the influential vagal drivers which contribute to maintaining AF [65].

The Vein of Marshall (VOM) within the Ligament of Marshall (LOM) contains another common target region for ablation procedures [50,53,66]. The VOM extends from the coronary sinus, between the left PVs and left atrial appendage, then traverses between the base of the left superior PV and pulmonary artery before attaching to the pericardium superiorly [67,68]. In this general region the VOM, myocardial sleeve and autonomic ganglia are found, with the ganglia located in a fat pad between the left PVs and left atrial appendage [67,68,69] Studies have shown that the LOM may act as a conduit of sympathetic innervation between the ventricles and the left superior ganglia [67]. In some patients, the LOM is an electrically active bypass of the left atrium/PV junction, rendering PV isolation ineffective [53].

There is growing interest in some of the more anatomically inaccessible GPs for ablation purposes. The Transverse Sinus GP (TSGP) resides between the pulmonary artery and base of the aorta, within the transverse sinus. It is sometimes referred to as the Great Artery GP in accordance with its associations [39]. The Aorto-caval or Superior Vena Caval-Aortic ganglion (SVC-Ao) is found along the posteromedial wall of the superior vena cava, the anterolateral wall of the ascending aorta and superior to the right pulmonary artery [70]. It is also referred to as the Superior Right (SR) GP. The SVC-Ao GP was believed to be a large, sprawling GP expanding from the anterolateral aspect of the aorta to the posterior portion of the aorta [71]. However, more recently it is suggested that two separate GPs reside in this area, the TS GP and the SVC-Ao GP. The TS GP and SVC-Ao GP are not as commonly targeted for ablation compared to other GPs and have mainly been targeted in canine studies [72,73] (Table 1). This is owing primarily to the difficulty in accessing them, with an epicardial approach preferred over an endocardial approach to avoid ablation within the great vessels [66]. The aorto-caval ganglion receives preganglionic parasympathetic innervation from the vagus nerve, while its postganglionic neurons send impulses to the atrium and superior vena cava. The SVC-Ao GP is believed to be the ‘head station’ for extrinsic cardiac ANS innervation to the heart [73]. Previous studies have shown this GP to shorten the effective refractory period and increase the window of vulnerability to arrhythmias at all atrial and PV sites influenced by stimulation of the vagal trunk [73]. It is also known to act as a trigger of SVC [73] (Figure 4). Hyperactivity of the SVC-Ao GP is also known induce ACh injection which acts as a trigger for SVC firing, but the exact mechanism is not entirely understood and requires further study [72,73] (Figure 4).

## 4. GP Ablation for AF

Therapeutic interventions for AF have been adapted to target the pathophysiological state involved in structural remodeling or to influence the excitation of ion channels and adrenergic receptors [21,74]. Many therapeutic approaches are employed to serve as a preventative measure, aiming to inhibit the primary occurrence of new-onset AF or secondary recurrence of AF, and are less effective in cases of persistent AF or AF with a prolonged duration [21,75,76]. Many invasive techniques have been adapted to target symptomatic drug refractory AF [77]. Surgical ablation such as the classical ‘cut and sew’ Cox-Maze procedures, claim a 97–99% success rate and have been deemed by some to be more effective than catheter-based approaches [78,79,80]. The drive to develop less aggressive techniques has encouraged the development of minimally invasive catheter-based procedures [81]. In the last 20 years, catheter cardiac ablation has become an established, fundamental treatment strategy for AF. Catheter ablation aims to relieve symptoms of AF, by eliminating the trigger of AF or altering the arrhythmogenic tissue underlying AF [80,82].

### 4.1. GP Localization and Its Importance for Effective Ablation

The active area of all GP sites, i.e., the GP areas where the vagal response is mostly elicited, appears significantly higher in AF compared to non-AF patients [83]. Additionally, the maximum R-R interval is significantly longer in AF patients after high frequency stimulation (HFS), when compared to non-AF groups [83]. Active GP areas are more prevalent on the right side of the PVs, with no considerable difference observed between paroxysmal, persistent and long-standing persistent AF patients [84,85,86,87] (Table 1). Ablation of active GPs on the right side of the PVs resulted in 92% sinus rhythm maintenance in patients at three month follow-up [86]. Zheng et al. reported that there are a mean of 2.8 active GP sites on the right side (Waterson’s grove and LOM) compared to 1.4 on the left side [84]. A reduced number of active GPs is associated with cardiac and neural remodelling and subsequent development of AF [84]. 95% of GPs are cholinergic and when activated a local release of ACh leads to bradycardia [62]. In chronic AF, there is a down regulation in the number of available ACh receptors, weakening the vagal response from GP areas upon stimulation [62]. This suggests that the strength of the vagal response is associated with a number of active GP areas before ablation, and higher numbers of GP sites ablated are significantly correlated with reduced AF recurrence at 12-month follow-up [84]. Similar findings are reported by others where 5 active GPs were identified on the right and 2.7 on the left side [88,89]. Again, this was directly linked to higher long-term success rates in patients with a mean number of active GPs over five [84].

A recent study by Hu et al. indicates that endocardial ablation of the right anterior GPs produced a significant increase in heart rate in 93% of patients [16]. In addition, there appears to be an essential role for the right anterior GP which inhibits positive vagal responses and increases heart rate during pulmonary vein isolation (PVI) [90]. These findings demonstrate the importance of GP ablation of specific sites between the PVs and interatrial groove when targeting AF. While ablation of right-sided GPs is a significant factor in minimizing and reducing AF recurrences, active GPs on the left have also been targeted for ablation in some studies (Table 1).

The modulation of SA and AV nodal function is governed by the extrinsic cardiac ANS. Animal studies have demonstrated that GPs on the right side act as “integration centers” and are capable of modulating the effect of stimulated left-sided GPs on AV and SA nodal responses [91]. HFS of the right inferior GPs has shown how they selectively innervate the AV node in humans [92]. As previously demonstrated in animal studies, ablation and mapping of right-sided GPs in humans with HFS can lower the number and magnitude of GP activity on the left side [93]. Neural pathways between left atrial GPs and the AV node have been shown to pass through the lower right GPs [94]. In support of this, there was significantly higher AF and atrial tachyarrhythmia (AT) recurrence rates reported in patients who underwent selective GP ablation of the right side first [93]. This study also implied that ablation of one active GP out of sequential pacing is insufficient for vagal denervation [95]. It is worth noting that no significant difference was observed between patients who underwent extensive anatomical mapping instead of HFS [93].

Despite our growing understanding of the anatomical map and location of GPs, the extent to which GPs are hyperactive and are viable targets for ablation is still largely unknown. GPs can be identified and targeted by applying HFS [44,96] or by ablating at presumed anatomic sites [97,98] (Table 1). The vagal response of GPs to HFS is found to be very specific, but the sensitivity of HFS in portraying the full extent of GPs is still lacking [52]. The first comparative study was carried out by Pokushalov et al. in which they demonstrated that the AF freedom rate after 13 month follow-up was 42.5% and 77.5% in HFS-induced and anatomical mapping ablation groups, respectively [97]. An explanation for this may be the greater extent to which ablation (RF ablation in this case) targets anatomical GP regions in comparison to specific HFS mapped areas. Synchronized HFS serves as an alternative method and involves the delivery of current during the atrial refractory period. This helps identify GP ablation sites based on the activation of autonomic neural elements and has been associated with better outcomes [94]. However, this technology is only applicable for patients in sinus rhythm and further research is required for its use for persistent AF. A case report study by Romanov et al. showed that using D-SPECTTM SUMO image acquisition after injection of I-mIBG provides a 3D cardiac electro-anatomical map that can be used to identify target sites [99]. This approach can potentially increase the efficacy of the ablation procedure by accurately identifying GPs that are verified by HFS [100]. Furthermore, GPs can be identified with additional complex fractioned atrial electrograms (CFAE) around the GP area [101,102].

An additional factor is that the efficacy of GP ablation can be diminished by the surroundings, e.g., the epicardial adipose tissue (EAT) and epicardial fat pads. The location and amount (>5 mm) of EAT may act as a protective covering of the coronary vessels to prevent vascular damage, serving as an insulating cushion to targeted epicardial sites, and minimizing the efficiency of thermal-based ablation strategies [103]. 80% and 100% of the major five anatomical GP sites are found and overlaid at CFAE sites and left atrium-EAT respectively [104]. GP ablation through positive vagal response by HFS stimulation on CFAE areas has shown sinus rhythm maintenance in 71% of patients with paroxysmal AF [102]. Extensive ablation of these CFAE areas defines the boundaries for GP ablation [102,104]. Left atrium-EAT and CFAE areas have similar distribution, and are adjacent to vagal response sites [101,104]. Ablation of the anterior fat pads has also been investigated, with many contradictory results recorded [105,106].

### 4.2. Technical Procedures for GP Ablation

RF ablation is a well-established technique for GP ablation and is employed in many ablation procedures (Table 1) [107]. Cryoablation is not as commonly used, despite being found to significantly reduce the surface area of GPs, with the SL and ARGPs most dramatically reduced overall [108]. However, with increased reports of its efficacy in treating AF, interest is growing in cryoablation as a stand-alone ablation modality and in the development of cryoablation devices [109]. Cryoablation can also be used in conjunction with RF to target GPs and achieve PVI [107,108]. One study presented a comparison between a group (35 patients) with PVI treated with cryoablation and an additional GP ablation and a group (65 patients) with only PVI, which showed comparable results of sinus rhythm maintenance at 94% and 89% at 12 months, respectively [107].

The extent to whether GP ablation alone contributes to successful abolition of AF is not clear and early experiments by Pokushalov et al. conducted using RF show freedom from AF in 77.5% of patients with paroxysmal AF [97]. A similar outcome was recorded in another of their studies that included 56 patients with paroxysmal AF which yielded a 71% success rate upon ablation of GPs [110]. Furthermore, the same group demonstrated that GP ablation alone over the course of 24 months resulted in freedom of AF in 38.2% of patients with persistent AF, in comparison to higher success rate in 12 months follow up studies [111]. This work also revealed that the success rate increased substantially (59.6%) after performing additional ablation procedures, suggesting that GP ablation may be most effective when accompanied by other ablative procedures such as PVI, rather than a single-shot approach. Interestingly, comparison of results from a single ablation procedure at 13 months and 24 months in two studies by the Pokushalov group showed success to be 77.5% and 38.2%, respectively. Patient numbers were similar in these two studies which may stand as a good comparison for incorporating the long-term effects of GP ablation; however, the types of AF did vary [97,111]. It is plausible that the different success rates may be influenced by the type of AF with a typically lower success related to persistent AF and a higher rate associated with paroxysmal AF [97,111].

PVI is associated with denervation of the ANS and a significant reduction in AF recurrence [82]. Most studies have incorporated PVI into their GP ablation procedures. Investigation of comparative studies of PVI and GP ablation alone or as combined procedures present intriguing results. Studies have shown that a stand-alone PVI yields higher success rates than GP ablation procedures alone [112,113]. However, in studies comparing PVI alone with PVI + GP, the success rate increases from anywhere between 20% and 28% in short-term follow-up of less than 12 months [114,115]. Success of PVI + GP ablation procedures can range from anywhere between 50% and 91% in studies involving all types of AF (paroxysmal, persistent and long-standing persistent) [116,117]. Higher success rates have been associated with paroxysmal AF patients in comparison to long-standing persistent patients at 86% and 50%, respectively [116]. However, some studies recorded high success rates in patients with persistent and long-standing AF, showing an incoherence between AF subtypes [118]. Typically, PVI with GP ablation are carried out in one session or in two stages, giving the patient time to recover between procedures. Hybrid procedures involving initial endocardial PVI followed by GP ablation at a later stage have been trialed on patients with persistent or long-standing persistent AF. The outcome of these procedures shows a high success rate of 93% and 82% at 12 month follow-up, respectively [119,120]. It is plausible that hybrid procedures may be more appropriate in treating these types of AF. The addition of PVI to GP ablation procedures increases the success rate regardless of AF type; however, more testing would be required throughout AF groups to delineate the most appropriate and efficient procedure.

The ‘mini-Maze’ procedure and Dallas lesion set are examples of adaptations that have been made to some procedures where epicardial PVI is incorporated into lesion sets, with promising results. RF energy is used as an adaption from the original Cox Maze ‘cut and sew’ methods. These methods can either intentionally or unintentionally integrate the ablation or intersection of GPs into their lesion sets [93]. Mini-maze procedures with intentional GP ablation has proved to be successful in treating AF in previous work. Outcomes recorded from two studies over sixteen months conducted by Onorati et al. and Matsutani et al. showed 83 ± 7.9% freedom from AF (75 patients) and 90% of (18) patients in sinus rhythm [118,121]. A Dallas lesion set modified from the Cox Maze III procedure also shows some potential for AF treatment [122]. A long-term two year follow-up has shown the Dallas lesion set to bring freedom from AF in 80.6% of patients with long-standing persistent AF [123,124]. These are similar data to those reported in Cox Maze studies that incorporated both paroxysmal and persistent AF in their study population [123]. It is possible that the extensive lesions formed during these procedures may in fact be important for treating particularly difficult and advanced AF cases and may inadvertently have included GPs in the lesions. Endocardial and epicardial access during PVI procedures have been associated with unintentional damage and incidental ablation at GP sites [125]. PVI via thermal epicardial approaches can result in overlap of ablation lesions with numerous GP sites, while the endocardial thermal approaches may induce collateral damage by conductive heating. For PVI with GP ablation, mapping can be used to locate gaps in ablation lines to test for electrical block in targeted areas [124]. Epicardial access for GP ablation with PVI yielded a rate of freedom from AF ranging from anywhere between 65% and 90% [121,126]. Similarly, the endocardial approach yielded 73.5%–91% freedom from AF [115,117]. Success rates involving GP ablation mainly appear to be similar, whether procedures are done via epicardial or endocardial approach.

### 4.3. GP Ablation for Non-AF Cardiovascular Conditions

GP ablation may offer an alternative way of treating other conditions that are related to an imbalance of cardiac ANS activity. Post operational AF (POAF) typically appears two–five days after cardiac surgery and can be associated with serious complications including cardiac failure, stroke and death [100]. Ablation of GP sites has been shown to significantly lower the incidence of POAF by 93% in a randomized controlled trial after coronary artery bypass grafting [88]. However, this approach is sometimes not clinically desirable. To avoid ablation with its destruction of anatomical structures and capacity for collateral damage, alternative measures have been examined, for example involving the neurotoxin Botulism produced by the bacterium Clostridium Botulinum [127]. Studies have shown that the intraoperative injection of Botulism toxin into epicardial fat pads can significantly reduce instances of POAF [127]. The neurotoxin temporarily blocks the exocytotic release of ACh and diminishes sympathetic and parasympathetic activity, highlighting the involvement of autonomic imbalance and GP activation in the mechanism of POAF. The effects of the Botulism toxin can last anywhere between one and six months and provide a better, untaxing alternative to the use of β-blocker medication. In studies by Pokushalov et al. and Romanov et al. the number of AF recurrences in patients administered Botulism injections showed a decrease of AF instances (7% Botulism group, 30% Placebo group, and 23.3% Botulism group, 50% Placebo group) at 12 and 36 months, respectively [127,128]. The Botulism toxin was found to induce a pronounced alteration of heart rate variability (HRV) in patients at six months, with heart rate parameters remaining significantly reduced during follow-up [127].

## 5. Discussion

The ablation of GPs appears to be an efficacious technique for improving outcomes of patients with paroxysmal, persistent and long-standing persistent forms of AF. Nonetheless, some very important questions remain unanswered. The long-term outcomes of GP ablation, the precise location and depth of GPs, and the exact mechanism in which GP ablation results in improved outcomes for AF are still not fully understood. Similarly, the ablation techniques used pose a risk of damaging the myocardium and surrounding structures.

Collateral damage is a significant drawback to current thermal ablation techniques. Cardiac tamponade, PV stenosis, oesophageal fistula and thrombi are among the associated risks with current ablation energies [1]. Another drawback to thermal ablation is the difficulty in delivering precise, appropriate energy to GPs. While GPs in association with PVs are accessed with relative ease, others are found in concealed locations. Overall, there is also significant complexity involved in catheter positioning from both within the pericardial space and the heart itself. Additionally a more efficient and effective visualization of GPs using imaging techniques such as SPECT^TM^ SUMO (Spectrum Dynamics Medical Limited, Caesarea, Israel) and I-mIBG may provide additional information for a much better localization before ablation [99]. These advances have significantly propelled research over the last decade. While the understanding of GP location is sometimes obscure, this may be due to the degree of anatomical variability between individuals [125]. However, the specifics in terms of report accuracy of the GPs that are targeted in some research papers remain ambiguous, with some studies not including nor clearly describing which GPs, or where they ablated [123,124]. This causes difficulty when comparing results from different studies targeting specific GPs associated with the maintenance of neural pathways and their subsequent effects on the SA and AV nodes [86,94]. Similarly, in some procedures with PVI + GP ablation, no clear reference is made to which GPs are ablated or whether there is an overlap of PVI lesions with targeted GP sites, making it challenging to compare success rates linked to GP ablation [120].

Augmented success rates (by 21%, 20% or 28%) with combined procedures is evident thus far only in short term follow-up (12 months or under) and with small patient numbers [61,115,118]. The added success of the PVI + GP ablation procedures in comparison to PVI alone is much lower (8%, 2.5% and 5%) in long-term follow ups (two–five years); however these studies include many more patients [107,114,129]. Due to the different techniques and study designs in clinical studies in the literature, it is difficult to assess and make a true comparison of success. HRV has been found to be a predictor of ablation success and is a useful, non-invasive tool for investigating cardiac autonomic tone [130,131]. HRV measures the fluctuations of time intervals between consecutive heartbeats [132]. An increased heart rate has been found to have positive associations with freedom from AF [130]. A recent study by Goff et al. showed a correlation between HRV in patients with paroxysmal AF who previously underwent PVI and the recurrence of AF [130]. While HRV is not always associated with PVI, an average increase of 60.6 ± 11.3 to 70.7 ± 12.0 beats per minute was recorded in 53% of patients at 12 months follow-up in this study [130]. Overall, it is evident that sufficient disruption of vagal responses results in an increased HR and freedom from AF. Coinciding with an adjusted HR is a shortening of AERP [133]. This has been found to facilitate the genesis and coexistence of numerous signals linked to AF [134]. Studies have shown the relationship between shortening of fibrillation intervals and AERP [36,135]. Additionally, the mean AERP has been reported to be shorter in persistent AF than those with paroxysmal AF due to electrical remodelling [136]. Reports by Lee et al. have also linked prolonged AERP with future development of AF with possible induction of remodelling over a twelve year follow-up [133,137]. Dispute remains over the relationship of action potential and refractory period in AF initiation, despite extensive animal and human studies [133]. Despite its importance, AERP is not a common parameter measured by clinicians. This may be owing to the technicalities associated with the recording of AERP in humans, in particular its inability to be recorded during AF [138].

In addition, it is possible that the positive post-procedural effects of GP ablation may only persist for a short amount of time. It may also be plausible that the GPs are not entirely ablated, enabling regeneration and the formation of new re-entrant pathways around the proximity of the GP, due to thermal myocardial damage caused by RF for example. Another reason for a low, long-term success rate may be the internal or external factors influencing remodeling of the heart over time. Concern exists regarding the proarrhythmic relationship between GP ablation without PVI. This approach carries the risk of inducing increased atrial parasympathetic and sympathetic innervation, coupled with a decreased atrial effective refractory period [139]. Similarly, selective GP ablation has been linked to the formation of macro-re-entrant atrial tachycardias which may be associated with autonomic reinnervation [139]. Animal studies have linked reinnervation at four weeks post-GP ablation with the selectivity of the regions targeted [140,141]. Therefore, further study must be carried out to understand what may or may not cause this relapse to AF and what changes can be made to increase the denervation time induced by GP ablation, and reduce the occurrence of pro-arrhythmia. Currently sample size is a major limitation in many studies with numbers ranging from individual case studies to research including up to 306 patients [142,143]. Evidently, variation in patient population will significantly influence success percentages, making it difficult to draw accurate comparisons.

Despite our evolving understanding of the physiology and success associated with GP ablation in AF treatment, the complications and challenges are not yet fully understood. Most patients involved in GP ablation procedures experience paroxysmal, persistent or long-standing persistent AF with some studies comparing all three [116]. Patients with symptomatic AF or AF associated with valvular disease are also included in research studies [95,144]. Similarly, investigations into specific AF types are not consistent, which leads to difficulty in assessing the extent to which GP ablation is effective. In a large randomized control during thoracoscopic surgery, there were no reported benefits of GP ablation in patients exhibiting advanced AF [114]. This may further suggest that the role of the ANS in the disease progression of AF may diminish over time [64]. Therefore, while varying degrees of AF have been examined, the true success of GP ablation for each type remains ambiguous. Nevertheless, from the expansive research and meta-analysis undergone on GP ablation, results show that it does give relief from AF in most cases, both initially and in the long term [145,146]. While much work is required to provide consistency between experiments, it is evident that the potential exists for significant advances in the treatment of AF through targeted ablation of GP sites.

## Figures and Tables

**Table 1:** Ganglionated plexi as main target for AF.

Author, Year	GP Sites Ablated	Localization of GPs	Additional Intervention	Number of Patients	Control Group	Method of Access	Follow Up Period (Max)	Outcome
Iso K et al., 2019	Ligament of Marshall (LOM,) SL, AR, IL, IR	High frequency stimulation (HFS)	Pulmonary vein isolation (PVI)	42	-	Endocardial	N/A	(1) R-R interval was longer in patients with AF. (2) More active GPs were found in patients with AF.
Garabelli P et al., 2018	SL, LI, RI, AR	HFS	PVI	18	-	Endocardial	1.8 ± 0.8 years	(1) 48% freedom from AF in GP ablation alone. (2) 74% freedom of AF in in GP ablation + PVI.
Budera P et al., 2018	LOM, SL, AR, IL, IR	HFS	PVI (Box lesion)	38	-	Epicardial	12 months	(1) 82% AF free using two-staged hybrid ablation of non-paroxysmal AF.
Bagge L et al., 2017	LA GP + LOM (if identified (96%))	HFS	PVI	42	-	Epicardial	12 months	(1) 76% AF free after 12 months.
Budera P et al., 2017	LA GPs	Anatomical	PVI (Box lesion)/cavotricuspid isthmus ablation	41	-	Epicardial/endocardial	507.2 ± 201.1 days	(1) 80% AF free without ADD/re-ablation in 2 staged hybrid procedure after 1.5 years. (2) 65% AF free with ADD/re-ablation at last follow-ups.
Barta J et al., 2017	SL, AR, IR, IL, LOM	HFS	Box lesion, R + L PV isolation, lesion of LA isthmus, resection of LAA + connecting lesion of appendage base with LSPV	35	65	Epicardial	12 months	(1) GP ablation alone showed 97.5% in NSR with ADD, 50% in NSR without ADD.
Suwalski G et al., 2017	SL, AR, IL, IR	HFS	PVI, LAA	34	-	Epicardial	3 months	(1) 85% success of GP detection based on preoperative heart rate.
Saini A et al., 2017	SL, AR, IL, IR, LOM	HFS	PVI	109	-	Epicardial	5 years	(1) 79.6% AF free without interventions (ADD, cardioversion, CA).
Baykaner T et al., 2017	SL, AR, IL, IR	HFS	N/A	97	-	Endocardial	N/A	(1) Sources of AF were found in: 47% at the SLGP site, 34% at ILGP, 14% at ARGP and 19% at IRGP sites.
Nagamoto Y et al., 2017	SL, AR, IR, IL	HFS	PVI	1	-	Endocardial	Not specified	(1) Inferior GP ablation itself did not eliminate PV potentials. (2) PVI had possibly helped eliminate PV potentials by cumulative effect.
Xhaet O et al., 2017	AR, IR, SL	HFS	PVI	20	-	Epicardial	N/A	(1) GPs are a mandatory link to the right vagus and AV node.
Romanov A et al., 2017	GPs of left atrium	HFS	N/A	1	-	Epicardial	6 months	(1) Using D-SPECTTM SUMO image acquisition created 3D cardiac electro-anatomical mapping system for GP ablation. (2) Patient was AF free with no ADD.
Takahashi K et al., 2016	LOM, SL, AR, IL, IR	HFS	PVI	40	-	Epicardial	18.6 months	(1) >80% Complex fractioned atrial electrograms (CFAE) overlay GP sites while 100% of epicardial adipose tissue (EAT) overlay GP sites.
Sharma P et al., 2016	10 sites each side	HSF + anatomical	Mini maze	67	-	Epicardial	4.5 ± 2.3 years	(1) Selective ablation of right GPs first is linked to higher rate of AF recurrence. (2) Lower number of GPs on the left side observed.
Antoine H.G. et al., 2016	SL, AR, IL, IR, LOM	HFS	PVI	117	123	Epicardial	12 months	(1) GP ablation alone did not reduce the AF occurrence during thoracoscopic surgery (70% vs. 68%).
Jiang et al., 2016	SL, AR, IL, IR	Anatomical	PVI	12	-	Epicardial	Not specified	(1) Majority of PV firing ceased after targeting GP outside of circumferential line or addition ablation along previous circular lesion.
Gelsomino S et al., 2015	SL, AR, IL, IR, LOM + Waterston groove	HSF	PVI (cox maze IV)	306	213	Epicardial	7 years	(1) GP ablations with or without cox maze IV showed no significant difference on AF recurrence NSR.
Sakamoto S et al., 2014	SL, AR, IL, IR, LOM	HSF + anatomical	Modified cox maze	30	-	Endocardial	3 months	(1) Most active GP sites were located in the Right PV antrum. (2) Anatomic GP ablation showed a greater decrease in sympathetic and parasympathetic tone.
Mamchur S et al., 2014	GPs of left atrium	Anatomical	PVI (Hybrid)	10	-	Epicardial & Endocardial	12 months	(1) 100% restoration of sinus rhythm with all patients free from arrhythmia in 6–9 months.
Zheng S et al., 2014	SL, AR, IL, IR,	HFS	PVI	89	-	Epicardial	60 months	(1) Single-procedure success rate is 56.3% for paroxysmal AF, 27.3% for persistent AF, and 25% for long-term persistent AF.
Katritsis D et al., 2013	SL, AR, IL, IR	Anatomic	PVI	242	-	Endocardial	24 months	(1) PVI group 56%, GP 48%, PVI + GP 74% maintained sinus rhythm/free from AF. (2) PVI + GP ablation is best strategy.
Kondo Y et al., 2013	SL, AR, IL, IR	HFS	PVI (Maze IV)	16	-	Epicardial	3 months	(1) 81% maintained NSR. (2) For those with active GPs, 92% maintained SR. (3) IRGP is an important pathway between other GPs and the AV node.
Malcolme-Lawes L et al., 2013	SL, AR, IL, IR	HFS	PVI (Cryoablation)	30	-	Endocardial	N/A	(1) Presence of a LA neural network with a common entry to the AV node via the IRGP.
Pokushalov E et al., 2013	SL, AR, IL, IR, LOM	HFS	PVI	132	132	Endocardial	36 months	(1) 34% of patients without GP ablation were in sinus rhythm. (2) 49% of patients with GP ablation were in sinus rhythm.
Kasirajan V et al., 2012	SL, AR, IL, IR	Not specified	PVI	118	-	Epicardial	12 months	(1) Additional ablation needed in 5% of patients. (2) 80% had freedom from AF after single procedure with no need for antiarrhythmics.
Santini M et al., 2012	Left atrial GPs and LOM		PVI	22	-	Epicardial	22 months	(1) Ablation was successful in 73% of patients. (2) Freedom from AF was 91% without ADD.
Calo L et al., 2011	Left & Right atrial GPs	HFSand Anatomic	N/A	34	-	Endocardial	19.7 ± 5.2 months	(1) AF recurred in 29% of patients with anatomic ablation and in 76% of patients with the selective approach.
Krul S et al., 2011	SL, AR	HFS	PVI (Hybrid)	31	-	Epicardial	12 months	(1) 86% AF free after 1 year follow up without use of ADD.
Lim P et al., 2011	SL, AR, IL, IR	HFS	N/A	12	-	Endocardial	N/A	(1) Direct link between activation of the intrinsic cardiac ANS and PV ectopy.
Katritsis D et al., 2011	SL, AR, IL, IR	HFS	PVI	34	33	Endocardial	5 ± 1.3 months	(1) PVI group had 54.5% recurrence rate and re-ablation rate of 21.2%. (2) PVI + GP group had 26.5% recurrence and 17.6% re-ablation.
Mikhaylov E et al., 2011	SL, AR, IL, IR	HFS and Anatomic	PVI	35	35	Endocardial	36 months	(1) Freedom from arrhythmia without drugs at 12 months was 54.3% for GP group and 74.3% for CPVI group. (2) Re-ablation was done in 17% of GP group.
Ware A.L et al., 2011	GPs of Left and Right atrium, LOM	HFS	PVI (Maze)	20	-	Epicardial	25 months	(1) 89% of patients were free of AF. (2) 79% were in NSR. 3) 11% were in a paced rhythm.
Lim et al., 2011	GPs of Left and Right atrium	HFS	N/A	25	-	Endocardial	N/A	(1) 16% reduction in AF cycle length was found in PV adjacent to HFS site. (2) 9% reduction at PV-atrial junction.
Pukushalov E et al., 2010	SL, AR, IL, IR	No specific mapping	N/A	56	-	Endocardial	12 months	(1) 71% of patients free from AF.
Pokushalov E et al., 2010	SL, AR, IL, IR	Anatomic	N/A	89	-	Endocardial	24 ± 3 months	(1) 38.2% freedom from AF after single ablation.
Pokushlov E et al., 2009	SL, AR, IL, IR and Active GP areas	Anatomic and HFS	N/A	80	-	Endocardial	12 months	(1) Recurrence of AF was 57.5% in selective and 22.5% in anatomic.
Po S et al., 2009	SL, AR, IL, IR	HFS	PVI	83	-	Endocardial	22 months	(1) GP ablation alone decreased incidence of spontaneous PV firing from 65.1% to 14.5%. (2) Freedom from AF after first procedure was 80%.
Ohkubo K et al., 2008	SL, AR, IL, IR	HFS	PVI	21	-	Endocardial	8 months	(1) 69% of patients free from AF.
Danik S et al., 2008	SL, AR, IL, IR	HFS	PVI	18	-	Endocardial	6 weeks	(1) Early AF recurrence in 22% of patients.
Onorati F et al., 2008	Left and Right PVs, LOM	HFS	PVI (Maze)	31	44	Epicardial	12 months	(1) Freedom from AF was higher in group with GP ablation (92.9±6.9%) when compared to group without (62.5 ± 6.9%).
Pokshalov E et al., 2008	SL, AR, IL, IR	Anatomic	N/A	58	-	Endocardial	7.2 ± 0.4 months	(1) Transient vagal bradycardia was seen in 93% of patients. (2) 86.2% of patients free from AF.
Matsutani N et al., 2008	GP around Waterson’s + left side + LOM	N/A	PVI	17	-	Epicardial	16.6 ± 5.7 months	(1) 90% of patients in NSR. (2) 85% weaned from AADs after 3 months.
Puskas J et al., 2007	LOM	HFS	PVI	1	-	Epicardial	6 months	(1) No AF revealed at months 1, 3 or 6. (2) Patient reported as no symptomatic AF.
Sherlag B et al., 2005	SL, AR, IL, IR	HFS	PVI	33	27	Endocardial	10 months	(1) 91% of patients were free from AF in PVI + GP ablation group, PVI alone group was 70%.

Normal sinus rhythm (NSR), Anti-arrhythmic drugs (ADD), left atrial (LA), left atrial appendage, (LAA), catheter ablation (CA), heart rate variability (HRV).
